# Preparations offered to workers in different food services: application of the score for qualitative assessment of preparations

**DOI:** 10.3389/fnut.2024.1354841

**Published:** 2024-07-25

**Authors:** Pietra Oselame da Silva Dohms, Lize Stangarlin-Fiori, Caroline Opolski Medeiros

**Affiliations:** ^1^Postgraduate Program in Food and Nutrition, Federal University of Parana, Curitiba, Brazil; ^2^Postgraduate Program in Food and Nutrition, Department of Nutrition, Federal University of Parana, Curitiba, Brazil

**Keywords:** prepared food, restaurant, food quality, public health, nutrition programs and policies

## Abstract

The quality of preparations offered in the workplace can vary according to the different segments of food services and may impact the health of the workers. This study aimed to qualitatively assess the food preparation offered to workers in from different food services. A total of 384 preparations were offered to workers in Curitiba City, Brazil. The preparations from three different segments of food services were evaluated: commercial (pilot study), non-commercial, and outsourced, selected for convenience. To identify the preparations, the nutritionist was interviewed, and the production process was monitored. The Score for Qualitative Assessment of Preparations (EAQP) was applied to evaluate the preparations, and they were classified according to their quality: high, intermediate, low, and very low quality. The chi-square and Kruskal–Wallis tests with *post-hoc* Least Significant Difference (LSD) Test were used. Most of the preparations were of high quality (72.9%), using mainly the unprocessed or minimally processed ingredients. The preparations offered by the non-commercial food service provider had a better mean quality score when compared to other food services (*p* < 0.01). This study outcome is essential to help food service professionals to decide and choose the ingredients used in the preparations.

## 1 Introduction

Food services, responsible for providing food outside the home, have significant importance in the workers’ food consumption (1, 2). Thus, the establishments that offer healthy and adequate food for these individuals can be ideal environments for their health promotion (3, 4).

Promoting health is a topic on the current agenda of health professionals, society, and government entities. Therefore, governments must plan, coordinate, and update public policy actions that contribute to the development of strategies to promote the human right to adequate food for the population (5, 6). In Brazil, the public policy aimed at providing access to food for the working class is the Worker’s Food Program (PAT) (7). This program aims to improve the nutritional quality of the food offered to Brazilian workers, guaranteeing them at least one main meal, while providing tax benefits to companies registered in the program.

By adhering to the PAT voluntarily, the company provides meals at the workplace, using its system of production (non-commercial food services), also known as self-management, or through outsourced food service, when a company specialized in food services is hired by an institution that provides food (7, 8). The workers can also have access to meals in commercial food services, which is open to the public and produces and sells meals directly to consumers, through meal vouchers (8).

The PAT-accredited food services must offer quality meals. However, for the PAT to remain a relevant program for the promotion of health of workers through the provision of quality meals, it is necessary to update the qualitative parameters. The parameters must take into account the current recommendations for healthy eating practices in the Dietary Guidelines for the Brazilian Population (5) and the Food and Agriculture Organization (9). Therefore, the consumption of unprocessed or minimally processed foods should be promoted, the consumption of processed foods should be limited, and ultra-processed foods should be avoided (5). This is recommended since the previous studies have shown an association between the consumption of ultra-processed foods and the rising prevalence of non-communicable diseases, including obesity, cardiovascular and metabolic disease, and cancer (10, 11).

Although previous studies have assessed the quality of meals offered and consumed by food service workers through quantitative and qualitative methods (4, 12, 13), to date, no study has evaluated the quality of individual preparations from different food services, considering the purpose and extent of industrial processing of ingredients (5, 9, 14).

Based on this, the Score for Qualitative Assessment of Preparations (EAQP) was recently created, following the standards of self-service buffets offered to workers in food services (8). This instrument aims to assess the individual preparations qualitatively (8), mainly considering the extent and purpose of the industrial processing of food ingredients (5, 9, 14). The EAQP enables researchers and professionals to assess the quality of preparations in different types of food service (8), including commercial and non-commercial, such as restaurants, fast food restaurants, cafeterias, and university restaurants.

The application of the EAQP in preparations offered to workers from different food services will help to know the quality of food offered to these individuals, based on the reality of different food service segments. This knowledge is important to provide support in updating the recommendations of healthy eating practices by public policies aimed at supplementing food for the working class. Therefore, this study aimed to qualitatively assess the preparations offered to workers in different food services.

## 2 Materials and methods

### 2.1 Type of study, ethics committee, and sample design

This was a cross-sectional observational study with multiple cases (15), carried out in Curitiba city, Parana, Brazil, and was approved by the Research Ethics Committee of the Federal University of Paraná (CAAE: n° 98205318.2.0000.0102). The participants signed an informed consent form.

The study was conducted on three different segments of food services registered in the PAT: commercial food service, non-commercial food service (self-management), and outsourced services. The food services were selected through convenience sampling.

Initially, a pilot study (SA-pilot) was conducted in the commercial food service to verify the established data collection procedures. This food service was selected considering its location and the inclusion criteria. The non-commercial self-management (SA-1) and non-commercial outsourced (SA-2) food services were selected from the report of active PAT beneficiary companies (16) and considering the inclusion criteria. The food services in each segment that first agreed to participate in the study were selected. The owners of the food service companies consented in writing to participate in the study.

For the selection of the three food services, the following inclusion criteria were used: being a small and medium-sized company; producing and distributing meals at lunchtime through a self-service buffet on-site; offering at least ten preparations, including salad, side dish, main course, and dessert; and offering food to PAT beneficiary workers. The three food services had nutritionists in charge of the place.

It should be noted that in Brazil, the nutritionist is the legally qualified professional to conduct the nutritional activities of the PAT, aiming to promote healthy food for workers (17).

### 2.2 Data collection

Data collection took place during the summer of 2018 and 2019. The study was conducted in the same season of both years to maintain the pattern of supplies of the preparations by the food services. This was done as there may be changes in the supply of preparations in the autumn and winter when temperatures are lower.

In SA-pilot, the data collection was conducted for five consecutive days, while in SA-1 and SA-2, it was conducted for 2 weeks, totaling 10 days of collection. The data collection of the preparations offered to workers at lunchtime from the SA-1 and SA-2 food services took place from Monday to Friday. The first 2 days were dedicated to observing and understanding the food service operations, and to interviewing the nutritionist. The other days were dedicated to monitoring the production process.

### 2.3 Interview with the nutritionist

To know the characteristics and the quality of each food service, the nutritionists were interviewed through a structured questionnaire. The questionnaire had five questions about professional data, number of employees, meals served daily for lunch, types of preparations offered, and the menu pattern (quantitative composition of preparations available to their customers in the buffet, such as salad, side dish, main course, and dessert). The nutritionists agreed to participate in the survey and answered the questions.

### 2.4 Follow-up of the productive process

The monitoring of pre-preparation and the preparation of the three food services was conducted to confirm the identity of ingredients and the quantities that were used in the preparations. All preparations (salad, main course, side dish I, side dish II, and dessert) offered in the self-service buffets were evaluated, and the ingredients were weighed in calibrated Explorer Ohaus^®^ scales, E0M210 model (Switzerland), and Ramuza^®^, IDR-7500 model (Santana de Parnaíba, Brazil). The salad preparations were defined as cold appetizers, usually available at the beginning of the self-service buffet (prepared with one or more types of vegetables, greens, or legumes, with or without seasoning). The main course was established as the preparation with the highest quantity of protein and consisted mainly of animal protein. The side dish I was a preparation based on cereals and legumes, such as rice and beans, and was offered daily on the menu. The side dish II consisted of vegetables, tubers, pasta, *farofa* (a kind of crumble), *polenta* (a meal prepared with corn), souffle, pies, and others. The dessert consisted of fruit or sweets.

### 2.5 Evaluation of the preparations

The EAQP (8) was applied by the researchers to qualitatively assess each preparation, considering the type of industrial processing of the ingredients used. The highlight of this method is the qualitative approach combined with scores by questions and an easy-to-interpret final score. The EAQP consists of 10 questions that assessed the classification of ingredients according to the extent and purpose of industrial processing: unprocessed and minimally processed (vegetables, roots, tubers, mushrooms, beans, lentils, cassava flour, pasteurized and ultra-pasteurized milk, natural nuts, meat, eggs, and coffee) (9), processed [food made with added salt, oil, sugar, or other substances from unprocessed or minimally processed food, elaborated by the industry and undergoing various preservation or cooking methods (14) – canned vegetables, nuts with added salt or sugar, canned fish in oil or water and salt, fruit in syrup, and cheeses (9)], and ultra-processed [products formulated mostly or entirely from substances extracted from foods or derived from food constituents (12) – soft drinks, powdered soft drinks, ice cream, chocolate, mayonnaise, industrialized meat broths, frozen and ready-to-heat products, sausages, hamburgers, and noodles] (9); the use of fresh or refrigerated fruits and vegetables as the main ingredient; the presence of low-fat meat (meat that has less than 10 g of total fat, 4.5 g of saturated fat, and less than 95 mg of cholesterol per 100 g serving) (18) or fish; the presence of whole grain and seeds; the presence of sugar, *rapadura* (sweet made from boiled sugarcane juice), honey, or molasses; and deep-frying by immersion in oil. The instrument’s answer options are yes, no, and not applicable (N/A), and the question scores range from four negative points to four positive points (Table 1).

**Table 1:** Score for qualitative assessment of preparations (EAQP) evaluated in food services.

Questions		Answer option		
		Yes	No	N/A
Q1)	Is the main ingredient of the preparation unprocessed or minimally processed?	4	−4	–
Q2)	If yes (Q1), is the main ingredient fresh or refrigerated fruit or vegetables?	1	0	0
Q3)	If no (Q1), are there any unprocessed or minimally processed ingredient(s) in this preparation?	1	0	1
Q4)	Do you use low-fat meat or fish?	1	0	1
Q5)	Do you use whole grains or seeds?	1	0	–
Q6)	Do you use sugar, *rapadura*, honey, or molasses as a cooking ingredient?	−1	1	–
Q7)	Is the preparation deep-fried?	−1	1	–
Q8)	Do you use processed ingredients?	−1	1	–
Q9)	Do you use ultra-processed ingredients?	−3	3	–
Q10)	If yes (Q9), is it the only ingredient in the preparation or do you use two or more ultra-processed ingredients in the preparation?	−3	0	0

Adapted by Dohms et al. (8). N/A, not applicable. – Answer option not included in the question.

The listing of ingredients and their respective quantities were recorded during the monitoring of the production process. Based on this, the EAQP was applied to all preparations offered by the food services. At the end of the instrument application, each preparation received a score that classified the preparation into four quality levels: high (score ≥ 11), intermediate (score 6 to 10), low (score 0 to 5), and very low (score ≤ −1) quality (8).

### 2.6 Data analysis

The data were analyzed using Statistica software version 7.0, and descriptive measures and frequency tables were used. The five types of preparations were analyzed according to what was desirable and undesirable, considering the response options according to the EAQP. In the answer options, Yes and No, for questions 1 to 9, it was considered a desirable answer option when the score was positive, and undesirable when the score was less than (−1 or − 4) or equal to zero. Question 10 was considered desirable when the score was equal to zero, and undesirable when the score was −3. For questions 2, 3, 4, and 10, which also presented Not Applicable as an answer option, it was considered desirable when this option was checked (Table 2).

**Table 2:** Questions analysis according to answers on desirability.

Questions	Yes	No	N/A
Q1) Is the main ingredient of the preparation unprocessed or minimally processed?	Desirable	Undesirable	–
Q2) If yes (Q1), is the main ingredient fresh or refrigerated fruit or vegetables?	Desirable	Undesirable	Desirable
Q3) If no (Q1), are there any unprocessed or minimally processed ingredient(s) in this preparation?	Desirable	Undesirable	Desirable
Q4) Do you use low-fat meat or fish?	Desirable	Undesirable	Desirable
Q5) Do you use whole grains or seeds?	Desirable	Undesirable	–
Q6) Do you use sugar, *rapadura*, honey, or molasses as a cooking ingredient?	Undesirable	Desirable	–
Q7) Is the preparation deep-fried?	Undesirable	Desirable	–
Q8) Do you use processed ingredients?	Undesirable	Desirable	–
Q9) Do you use ultra-processed ingredients?	Undesirable	Desirable	–
Q10) If yes (Q9), is it the only ingredient in the preparation or do you use two or more ultra-processed ingredients in the preparation?	Undesirable	Desirable	Desirable

N/A, not applicable. – Answer option not included in the question.

The chi-square test was applied to assess the differences in the quality of preparations between the food services. The Kruskal–Wallis test with *post-hoc* Least Significant Difference (LSD) Test was used to verify the difference in the EAQP classification score and the level of processing of the ingredients in the preparations. The significance level considered for analysis was *p* < 0.05.

## 3 Results

A total of 384 preparations were evaluated in the three food services, 24.2% (*n* = 93) in SA-pilot, 30.5% (*n* = 117) in SA-1, and 45.3% (*n* = 174) in the SA-2. Most of the preparations (35.4%) consisted of salad, distributed among the food services in the following proportions: 44.1, 37.6, and 29.3%, respectively (Table 3).

**Table 3:** Various preparations of different food services evaluated during the production process and their menu structure.

Preparations	Analyzed preparations								Menu structure		
	Total		SA-pilot		SA-1		SA-2		SA-pilot	SA-1	SA-2
	*n*	%	*n*	%	*n*	%	*n*	%	*n*	*n*	*n*
Salad	136	35.4	41	44.1	44	37.6	51	29.3	13 to 14	5 to 6	6 to 7
Main course	45	11.7	11	11.8	11	9.4	23	13.2	3 to 4	1	2 to 3
Side dish I	64	16.7	9	9.7	23	19.7	32	18.4	3	2 to 3	4
Side dish II	81	21.1	21	22.6	17	14.5	43	24.7	7	2	5 to 6
Dessert	58	15.1	11	11.8	22	18.8	25	14.4	3 to 4	2 to 3	3
**Total**	384	100	93	100	117	100	174	100	29 to 32	12 to 15	20 to 23

Salad: cold appetizers, usually available at the beginning of the self-service buffet. Main course: preparation with the highest offer of protein and consisted mainly of animal protein. Side dish I: cereals and legumes, such as rice and beans. Side dish II: vegetables, tubers, pasta, farofa, souffles, pies, among others. Dessert: fruit or sweets. SA-pilot: commercial food service, pilot study. SA-1: non-commercial self-management food service. SA-2: non-commercial outsourced food service.

Furthermore, it was found that the menu pattern was different in the three food services. The SA-pilot had the superior menu pattern, the SA-2 had the intermediate pattern, and the SA-1 had the basic pattern, and they served an average of 222 (±6.06), 449 (±25.90), and 234 (±64.34) daily lunches, respectively. In addition, the number of meals offered daily varied among the food services (Table 3).

The quality of preparations was independently evaluated according to the EAQP, not considering the type of food service. It was found that most preparations (72.9%) were classified as high quality (Table 4). Most of the salad (92.7%) and side dish I (100%) were classified as high-quality preparations (Table 4), and these preparations used unprocessed (94.9%) or minimally processed food as the main ingredient (100%) (Table 5).

**Table 4:** Classification of the quality of the preparations evaluated according to the score for qualitative assessment of preparations (EAQP).

Preparations		Quality classification of preparations according to the EAQP							
	Total	High		Intermediate		Low		Very low	
	*n*	*n*	%	*n*	%	*n*	%	*n*	%
**Total**	384	280	72.9	20	5.2	35	9.1	49	12.8
**Subgroups**									
Salad	136	126	92.7	0	0	4	2.9	6	4.4
Main course	45	19	42.2	9	20	9	20	8	17.8
Side dish I	64	64	100	0	0	0	0	0	0
Side dish II	81	36	44.4	11	13.6	17	21	17	21
Dessert	58	35	60.4	0	0	5	8.6	18	31

Salad: cold appetizers, usually available at the beginning of the self-service buffet. Main course: preparation with the highest offer of protein and consisted mainly of animal protein. Side dish I: cereals and legumes, such as rice and beans. Side dish II: vegetables, tubers, pasta, farofa, souffles, pies, among others. Dessert: fruit or sweets. EAQP: Score for Qualitative Assessment of Preparations. High quality (score ≥ 11), intermediate quality (score 6 to 10), low quality (score 0 to 5), and very low quality (score ≤ −1).

**Table 5:** Frequency of answers to the questions according to the score for qualitative assessment of preparations (EAQP).

Assessment by EAQP	Salad				Main course				Side dish I				Side dish II				Dessert			
	Desirable		Undesirable		Desirable		Undesirable		Desirable		Undesirable		Desirable		Undesirable		Desirable		Undesirable	
Questions	*n*	%	*n*	%	*n*	%	*n*	%	*n*	%	*n*	%	*n*	%	*n*	%	*n*	%	*n*	%
Q1	94.9	94.9	7	5.2	37	82.2	8	17.8	64	100	0	0	63	77.8	18	22.2	40	69	18	31
Q2	79.4	79.4	28	20.6	0	0	45	100	0	0	64	100	30	37.0	51	63.0	38	65.5	20	34.5
Q3	97.8	97.8	3	2.2	44	97.8	1	2.2	64	100	0	0	75	92.6	6	7.4	45	77.6	13	22.4
Q4	99.3	99.3	1	0.7	40	88.9	5	11.1	64	100	0	0	75	92.6	6	7.4	58	100	0	0
Q5	5.9	5.9	128	94.1	1	2.2	44	97.8	19	29.7	45	70.3	2	2.5	79	97.5	0	0	58	100
Q6	100	100	0	0	41	91.1	4	8.9	64	100	0	0	77	95.1	4	4.9	50	86.2	8	13.8
Q7	100	100	0	0	41	91.1	4	8.9	64	100	0	0	77	95.1	4	4.9	58	100	0	0
Q8	97.1	97.1	4	2.9	38	84.4	7	15.6	64	100	0	0	56	69.1	25	30.9	56	96.6	2	3.5
Q9	93.4	93.4	9	6.6	24	53.3	21	46.7	64	100	0	0	40	49.4	41	50.6	35	60.3	23	39.7
Q10	95.6	95.6	6	4.4	31	68.9	14	31.1	64	100	0	0	58	71.6	23	28.4	39	67.2	19	32.8

Q1) Is the main ingredient of the preparation unprocessed or minimally processed?; Q2) If yes (Q1), is the main ingredient fresh or refrigerated fruit or vegetables?; Q3) If no (Q1), are there any unprocessed or minimally processed ingredient(s) in this preparation?; Q4) Do you use low-fat meat or fish?; Q5) Do you use whole grains or seeds?; Q6) Do you use sugar, rapadura, honey, or molasses as a cooking ingredient?; Q7) Is the preparation deep-fried?; Q8) Do you use processed ingredients?; Q9) Do you use ultra-processed ingredients?; Q10) If yes (Q9), is it the only ingredient in the preparation or do you use two or more ultra-processed ingredients in the preparation? Questions 1 to 9, it was considered a desirable answer option when the score was positive, and undesirable when the score was less than or equal to zero. Question 10 was considered desirable when the score was zero and undesirable when the score was − 3. For questions 2, 3, 4, and 10, which also presented the option not applicable as an answer, it was considered desirable when this option was checked.

The main course and side dish II were the only preparations mostly classified as intermediate quality (20 and 13.6% respectively) and as low quality (20 and 21% respectively) (Table 4). These preparations, classified as intermediate quality, used some processed ingredients (15.6 and 30.9%), and approximately half of the preparations used ultra-processed ingredients (46.7 and 50.6%) (Table 5). It should be noted that most of the main courses (88.9%) used low-fat meat or fish (Table 5). Regarding the low-quality classification of the main course and side dish II (Table 4), it was observed that 31.1 and 28.4%, respectively, used more than two ultra-processed ingredients (Table 5).

The dessert preparation was mostly classified as very low quality (31%) (Table 4), as it used at least one ultra-processed ingredient or used an ultra-processed ingredient as the only ingredient in the preparation (32.8%), such as gelatin powder (Table 5).

The response frequency analysis of the EAQP questions indicated a low percentage of the use of whole grains and seeds in the preparations (Table 5). However, it is worth noting that despite 70.3% of side dishes I not using these ingredients, all food services offered brown rice daily at the self-service buffet.

**Figure 1:**
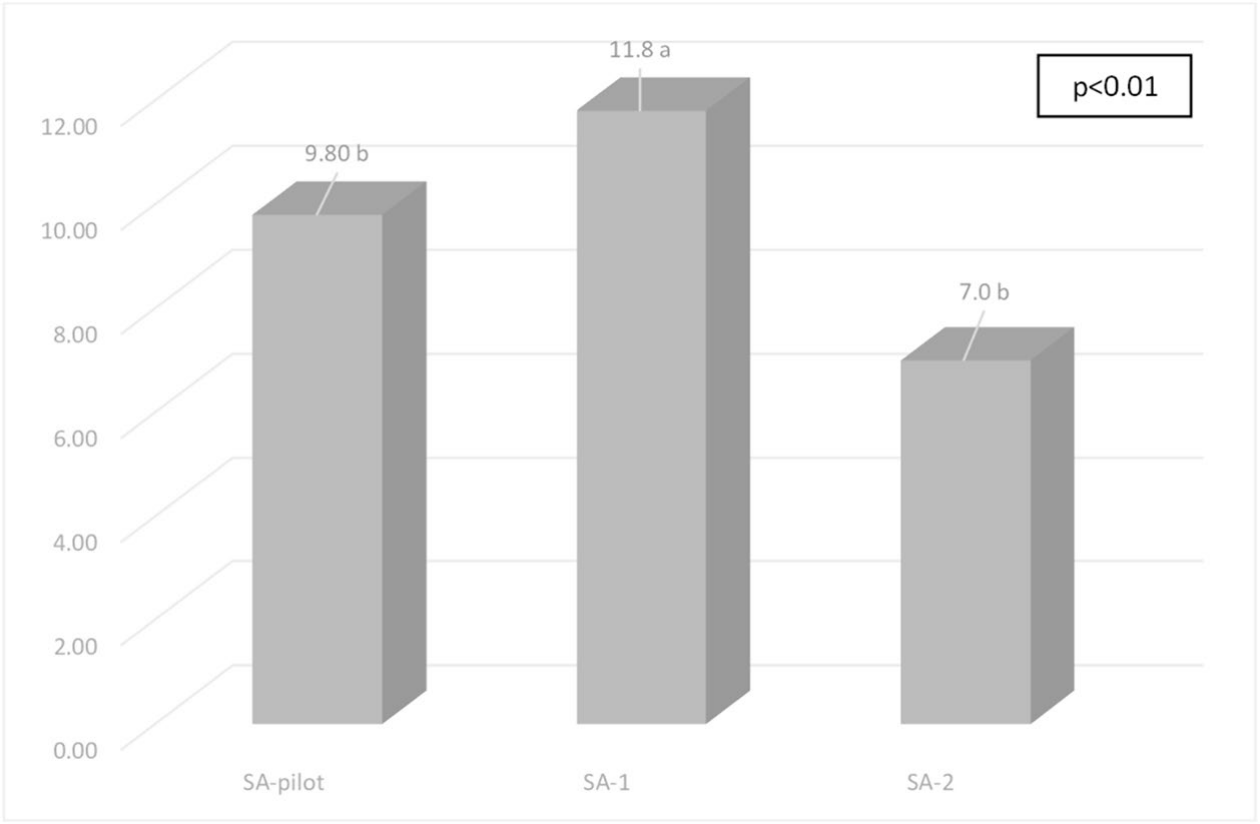
Mean score of the quality of the preparations offered in the food services, according to the score for qualitative assessment of preparations (EAQP). SA-pilot: commercial food service, pilot study, SA-1: non-commercial self-management food service, SA-2: non-commercial outsourced food service. High quality (score ≥ 11), intermediate quality (score 6 to 10), low quality (score 0 to 5), and very low quality (score ≤ −1). Chi-Square Test, *p* = 0.00001. Distinct letters correspond to the significant difference; *p* < 0.05; Kruskal-Wallis with *post hoc* LSD.

When comparing the food services, according to the mean of the EAQP score, it was evident that the SA-1 (11.8 ± 2.91), with a basic menu pattern, presented better quality performance of the preparations as compared to the other food services, which showed an intermediate (SA-2: 7.0 ± 7.74) and higher menu pattern (SA-pilot: 9.8 ± 5.39) (*p* < 0.01) (Figure 1).

**Figure 2:**
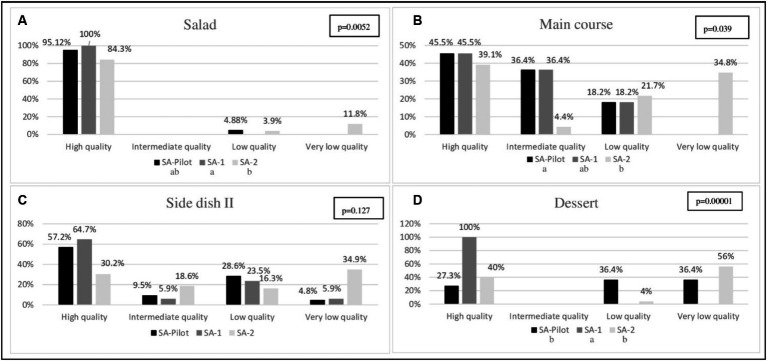
Classification of the score for qualitative assessment of preparations (EAQP) by type of preparation and the food service. **(A)** Salad, **(B)** Main course, **(C)** Side dish II, **(D)** Dessert. SA-pilot: commercial food service, pilot study, SA-1: non-commercial self-management food service, SA-2: non-commercial outsourced food service. High quality (score ≥ 11), intermediate quality (score 6 to 10), low quality (score 0 to 5), and very low quality (score ≤ −1). Kruskal-Wallis test, supplemented by the LSD test, *p* < 0.05. Distinct letters correspond to the significant difference. All preparations characterized as a follow-up, in the three food services, were classified as high quality.

The final classification of the quality of the preparations, by type and the food service, is illustrated in Figure 2. Although most salad preparations were classified as high quality (Figure 2A), there was a statistically significant difference (*p* = 0.0052) between these preparations among the evaluated food services. In SA-2, 11.8% (*n* = 6) of the salads were classified as very low quality. In addition, it was noted that SA-2 main course preparations were mostly classified as very low quality (*p* = 0.039) (Figure 2B). Side dish II was the only preparation that showed similarity between the food services evaluated (*p* = 0.127) (Figure 2C). However, there was a significant difference (*p* = 0.00001) in the quality of dessert offered between the food services (Figure 2D). The SA-1 offered 100% of the desserts classified as high quality, while the SA-pilot and SA-2 services had the highest number of desserts classified as very low quality (36.4 and 56.0% respectively) (Figure 2D). All preparations characterized as follow-ups in the study were classified as high quality; therefore, the comparison between food services was not performed.

## 4 Discussion

This study applied the EAQP instrument to preparations offered to Brazilian workers benefiting from the PAT in different food services segments, considering the restaurant management models often used in Brazil. This research suggests that a review of food preparations is necessary, with a focus on the industrial processing of ingredients. The public policies regarding food to be provided to workers must consider the quality of the meals.

The EAQP is a method of qualitative assessment of the preparations, which mainly considers the use of ingredients, according to their extent and purpose of the industrial processing (8). To assess the quality of food offered to workers, a qualitative approach should be considered, including the current recommendations for healthy eating practices, focusing on food choices (5, 19) used in the preparations that make up the menu of food services.

Most of the preparations offered to Brazilian workers in this research were classified as high-quality (72.9%) preparations. The main ingredient of these preparations was unprocessed or minimally processed food. This is positive for the health and eating habits of individuals (20, 21) and follows the current recommendations for healthy eating practices (5, 9, 19). Some preparations classified as high quality had some processed food as an ingredient but never used ultra-processed food as an ingredient (8).

The varied and daily consumption of unprocessed or minimally processed foods is recommended for a healthy and adequate diet, as it helps to prevent chronic non-communicable diseases (10, 11, 22–24). Furthermore, the increased consumption of these foods improves the quality of the population’s diet, as demonstrated by Moubarac et al. (25) when analyzing the dietary patterns of 33,694 Canadians.

When considering the type of preparation, salads were the most offered, and most were classified as high quality, as they were mainly made with unprocessed or minimally processed ingredients. Considering the relevance of the consumption of these foods, it is highlighted that making fruits and vegetables available in food services at the workplace is a favorable strategy to increase the consumption of these foods by workers (26).

However, SA-2, an outsourced food service, offered salad options that were rated as very low quality. These preparations, classified as very low quality, did not use unprocessed or minimally processed foods as the main ingredient and frequently used many ultra-processed foods (8). This shows a possible relationship with the daily offer of sauces to season the salads, such as tartar sauce, rosé sauce, or mayonnaise. These sauces were prepared on-site and offered at the self-service buffet to be eaten with the salads. In addition to the dressing option to season salad, the SA-2 also provided olive oil, salt, and vinegar. It should be noted that, for this study, only the sauce prepared in the food service was evaluated, and not the other seasonings available to the consumers.

The base of most sauces was soybean oil, a processed culinary ingredient, and the sauces had additional ultra-processed ingredients such as dairy compound, mustard sauce, and ketchup. Soybean oil is commonly used in food services for cooking, emulsifying, roasting, sautéing, and frying. However, this ingredient should be used in moderation, to avoid being harmful to health (27). A recommended alternative to improve nutritional quality, considering the nutritional composition of this preparation, is to replace soybean oil with extra virgin olive oil. This change will not change the classification of the quality of the preparation according to the EAQP, however, the consumption of extra virgin olive oil is associated with the prevention and lower risk of cardiovascular diseases (28) and type 2 diabetes (29).

Furthermore, the use of ultra-processed ingredients must be avoided. These foods, manufactured by the industry, contain added fats, salts, sugars, proteins, starches, and other substances rarely used in food service preparations, such as modified starches, hydrogenated or interesterified oils, and other artificial additives (14, 25, 30). The high consumption of ultra-processed ingredients can harm workers’ health, being associated with overweight and obesity (31, 32), non-communicable chronic diseases (33), depression (34), and other causes of mortality (35).

In addition, the ultra-processed ingredients are highly energetic. It is important to consider that the quality score provided by the EAQP instrument demonstrates a correlation with the caloric contribution of ingredients, depending on the extent and purpose of their industrial processing during preparation. As the proportion of calories originating from ultra-processed and processed ingredients increases, the overall quality of the preparation decreases (8).

It should be noted that the ingredients used in the preparations determine the quality of the preparation. Therefore, it is suggested to replace the ultra-processed ingredients in the preparations with minimally processed alternatives, such as cow’s milk instead of the dairy compound.

All options of side dish I were found to be of high quality, considering that they consisted of only minimally processed ingredients in the three food services. This information becomes relevant because this type of preparation, represented mainly by rice and beans, is present in the daily diet of the Brazilian population, and is the most consumed food among Brazilians (36). Furthermore, the side dish I was the dish that used whole grain the most, due to the daily offer of whole grain rice in food services. Incorporating whole grains and seeds in preparations is important for its potential to enhance the evaluation of these dishes in food services. These ingredients are valuable due to their well-documented health benefits, which include antioxidant properties, cancer prevention, cholesterol reduction (37, 38), decreased risk of heart disease, and support for weight management (39). The whole grains and seeds add significant nutritional value, being rich in fiber, vitamins, minerals, and antioxidants (40, 41). Furthermore, they can improve the flavor and texture of dishes, offering a richer and more satisfying sensory experience for consumers (42), thereby broadening the variety of options available.

The side dish II was the dish that used the most processed ingredients, such as pickled food, mozzarella cheese, parmesan cheese, tomato purée, and breadcrumbs. Most of these ingredients have salt or sugar added during manufacturing by the food industries. In addition, they are used in combination with unprocessed or minimally processed foods; however, their use should be limited (5, 9). These recommendations are in line with the American Heart Association, which advises reducing the consumption of foods rich in sodium, as it can contribute to reduced mortality from cardiovascular diseases (41).

Furthermore, considering the evaluation of the quality of side dish II, it was observed that most were classified as low or very low quality, as more than half of the preparations used ultra-processed ingredients and some were deep-fried.

The deep-fried preparations are usually included in the menu, as they are considered quick preparations and they have pleasant sensory aspects (43). In this sense, SA-2 stands out, offering French fries or fried *polenta* at least twice a week, as it was a contractual request between the contracting company and SA-2.

It is important to highlight that frequent consumption of deep-fried dishes and ultra-processed sauces in side dishes and salads can lead to various negative health consequences, such as obesity and weight gain (23, 40). Deep-fried dishes can increase the intake of trans fats and other harmful compounds formed during the process of frying. These compounds have been associated with an increased risk of cardiovascular diseases and type 2 diabetes (29).

In Brazilian food services, the main course is lunch, which is the meal with the main source of protein. The main course can consist of animal or vegetable protein. However, when animal protein is used, it must follow international recommendations in prioritizing low-fat meat or fish (23, 24).

It was shown in the present study that most of the main courses were made with low-fat meat. This brings positive benefits, as these foods generally have low levels of saturated fat content (23), are rich in high-quality proteins, provide all essential amino acids for health, and are important sources of vitamins and minerals. In addition, fish are an excellent source of omega-3 fatty acids, which are favorable for cardiovascular and brain health (44, 45).

However, the quality classification of these preparations was hindered by the presence of an ultra-processed ingredient (sausages, mustard sauce, soybean oil, or dairy compound). This demonstrates the importance of considering not only the type of meat used in the preparations but also, the other ingredients added to these preparations.

When comparing the different food services regarding the use of ultra-processed ingredients in their preparations, it was noted that SA-2 was the only food service that offered a very low-quality main course. This was mainly due to the inclusion of main courses featuring sausages, such as pepperoni sausage, or seasoned beef, chicken, and pork. These items contain additives and substances that change the color, flavor, aroma, and texture, thereby classifying the ingredients as ultra-processed (9, 14). Although these food options are considered practical and cheap for food services and generally have good acceptance by diners, studies demonstrate the negative impacts of these ultra-processed ingredients on the health of individuals (35, 46, 47), such as the rising prevalence of non-communicable diseases (10, 11).

Regarding the desserts offered, most were classified as high quality. This was mainly due to the daily offer of fruits as dessert, following international recommendations aimed at healthy eating (23). However, in SA-pilot and SA-2, 36.4 and 56% of the desserts were classified as very low quality, respectively. These food services used ultra-processed ingredients to prepare other desserts (such as dairy compound, gelatin, or industrialized biscuits), which is not in line with the recommendations of the food guide, that the ultra-processed desserts should be replaced with homemade fruits and sweets (5).

Therefore, it is important to increase awareness and educate professionals and workers about healthy food choices. The food services should review their menus to prioritize healthy options for workers and to align with public policies to promote health and healthy eating. This includes a preference for fresh and minimally processed ingredients in desserts (8). Furthermore, internal policies can be adopted to encourage the consumption of desserts that align with the nutritional guidelines. It is equally important to provide training to professionals in using healthy preparation techniques and the substitution of ultra-processed ingredients. Conducting awareness campaigns and including attractive options for healthy desserts on menus can encourage healthier choices and improve the quality of desserts in food services.

When comparing the different evaluated food services, the SA-1, non-commercial food service, had the highest mean EAQP score (11.8), even though it provided the fewest daily meals. SA-1 stood out for offering the most high-quality preparations, as it primarily used unprocessed or minimally processed ingredients. This finding suggests a potential connection between cost management in food services and the procurement of these ingredients. The commercial food services and non-commercial outsourced food services aim for profit, which may lead them to frequently use processed and ultra-processed ingredients in food preparations. This tendency was particularly evident in food services that had a higher number of daily preparations. It is possible that the procurement of these ingredients is related to the provision of long shelf life, high palatability (48), requests or satisfaction of the contracting party and target audience, raw material and employee cost management, agility in preparation time of these ingredients, and convenience (49).

The present study findings contributed to qualitatively assessing the preparations offered to the group of Brazilian workers, considering the extent and purpose of the industrial processing of food ingredients. However, the study has limitations. The study, with multiple cases, was carried out on three food services selected for convenience; therefore, it was not possible to make general statements about the quality of the preparations offered to workers benefiting from the PAT. The other food services may have different qualities from the food services evaluated in this study.

Furthermore, as data collection took place during the summer period, it is possible that, in other seasons of the year, food services use different ingredients in their preparations, due to the seasonal availability of the food. However, as the EAQP assesses the use of ingredients according to the extent and purpose of industrial processing, the change in season of the year will not change the classification of foods, and thus, the classification of the quality of preparations. Despite the limitations, it is considered an innovative study, which compared the quality of preparations in different food services.

Furthermore, although the EAQP does not provide an overall score for the menu, the assessment of individual preparations can be used to plan the menus with healthier food preparations. Therefore, attention should be paid to the procurement of ingredients used in the preparations, prioritizing ingredients with minimal processing, improving the quality of the preparations offered, and promoting the health of the workers. This can encourage consumers to make better food choices as the choice of food depends on what is offered at the meal buffet.

## 5 Conclusion

Most of the preparations offered to the workers from the various evaluated food services were classified as high quality, as they mainly used unprocessed or minimally processed ingredients as a base; this information is a positive aspect. However, the main course, side dish II, and above all, the dessert preparations, evaluated in food services, need improvements in terms of the quality of ingredients, as many were classified as low and very low quality. The food service with the best quality of preparations was SA-1, which used unprocessed or minimally processed ingredients as a base for all preparations and rarely used processed or ultra-processed ingredients. The differences in food services emphasize the need for public policies for providing food to the workers.

## Data availability statement

The original contributions presented in the study are included in the article/Supplementary material, further inquiries can be directed to the corresponding author.

## Ethics statement

The studies involving humans were approved by Research Ethics Committee of the Federal University of Paraná. The studies were conducted in accordance with the local legislation and institutional requirements. The participants provided their written informed consent to participate in this study.

## Author contributions

PS: Methodology, Validation, Formal analysis, Investigation, Writing – original draft. LS-F: Methodology, Validation, Supervision, Writing – review & editing. CM: Conceptualization, Formal analysis, Funding acquisition, Methodology, Supervision, Validation, Writing – original draft, Writing – review & editing.
