# Supplementary material for: Preparations offered to workers in different food services: application of the score for qualitative assessment of preparations
Source: Front Nutr. 2024 Jul 25;11:1354841. doi: 10.3389/fnut.2024.1354841 (PMC11307149; doi:10.3389/fnut.2024.1354841)
Supplement: Supplementary file 1 [file Image_1.pdf]

Preparations offered to workers in different food services

Materials and methods

SIMPLE DESIGN AND DATA COLLETION

DURING SUMMER

Food services from different segments

- Commercial
- Non-Commercial Self-management
- Non-Commercial Outsourced

INTERVIEW WITH NUTRITIONIST

FOLLOW-UP OF THE PRODUCTIVE PROCESS

EAQP

PREPARATION QUALITY CLASSIFICATION:

High Intermediate
Low Very low

DATA ANALYSIS

Results

FOOD PREPARATION

n=384

72,9%
HIGH QUALITY

EAQP

Final preparation quality classification

92% 100%
High quality

17.8% 31% 21%
Very low quality

Non-commercial self-management food service had a better mean quality score (p<0.01).

Conclusion: Most of the preparations offered to workers by food services, especially the non-commercial, were classified as high quality, as they mainly considering the type of ingredients with a lower level of processing.
